# Incidence of medically attended Lyme borreliosis across endemic regions of six European countries (BOLD): a prospective multicentre active surveillance study

**DOI:** 10.1016/j.lanepe.2026.101907

**Published:** 2026-10-26

**Authors:** Kate Halsby, Alexandra Loew-Baselli, Anna Moniuszko-Malinowska, Franc Strle, Johan Sanmartin Berglund, Ye Tan, Katherine T. Schneider, Mark W. Cutler, Warren V. Kalina, Raphael Simon, Joppe Hovius, Alje P. van Dam, Brad D. Gessner, Frederick J. Angulo, Juanita Edwards, Andreas Pilz, Elizabeth Begier, James H. Stark, the BOLD Study Group, Lenka Dybova, Miroslav Pavlasek, Daniela Verdanova, Joachim Weimer, Christine Kosch, Kai Mehltretter, Hans-Detlev Stahl, Viliam Cibik, Dagmar Zakova, Barbara Nieradko-Iwanicka, Dorota Bielska

**Affiliations:** a Pfizer Ltd, Tadworth, Surrey, United Kingdom; b Pfizer Corporation Austria GmbH, Vienna, Austria; c Department of Infectious Diseases and Neuroinfections, Medical University of Białystok, Poland; d Department of Infectious Diseases, University Medical Centre Ljubljana, Ljubljana, Slovenia; e Department of Health, Blekinge Institute of Technology, Karlskrona, Sweden; f Pfizer Inc, Cambridge, MA, United States; g Pfizer Vaccines, Pearl River, NY, United States; h Amsterdam University Medical Centers (UMC), Amsterdam, the Netherlands; i Pfizer Inc, Collegeville, PA, United States; j Pfizer Biopharma Group, Dublin, Ireland

**Keywords:** Lyme borreliosis, Lyme disease, Incidence, Surveillance, Europe, Burden of disease

## Abstract

**Background:**

Lyme borreliosis (LB) is endemic in many European countries. Comparing LB incidence between countries is challenging because case definitions and surveillance methodologies are inconsistently implemented. The prospective, epidemiological Burden of Lyme Disease (BOLD) study used active surveillance to characterise LB burden in Europe.

**Methods:**

LB patients were evaluated at 14 healthcare practices in high LB incidence areas in six European countries based on EUCALB-derived case definitions using standard of care (SOC) laboratory diagnostics (April 2021–December 2022). Enrolled cases were interviewed for medical/care histories and underwent additional standardised laboratory testing. OspA types were determined by in-silico sequence analysis where possible.

**Findings:**

Among participating sites, 797 LB cases were identified using SOC diagnostics. The incidence (per 100,000 population per year) of LB was 657 for 2021 and 661 for 2022. Incidence was highest in the Slovakian sites (2021, 3059; 2022, 1860) and lowest in the Polish (2021, 209) and German sites (2022, 75). When standardised laboratory testing was considered for disseminated cases, incidences remained relatively consistent for 5 of the 6 countries. *Borrelia afzelii* (serotype 2) was identified in 30/33 (91%) isolates.

**Interpretation:**

LB incidence was high in this prospective epidemiological study spanning multiple countries. The heterogeneity in LB incidence across regions illustrates inherent difficulties in aggregating estimates and highlights a need for robust regional monitoring and reporting, and standardised methods. Public health interventions for the prevention of LB should be considered.

**Funding:**

This analysis was supported and jointly funded by Valneva and Pfizer Inc. as part of their co-development of a Lyme Disease vaccine.

**Research in context**

**Evidence before this study**

We searched PubMed for studies describing the incidence of Lyme borreliosis (LB) in Europe from 2010 to Mar 24, 2026, using the key words (“Lyme disease” OR “Borreliosis”) AND “incidence” AND “Europe” without language restriction. Of 122 papers identified, 21 reported data on the incidence of LB in Europe: 17 with primary data, and four systematic literature reviews (SLR).

Six of the primary data studies only reported data from passive national surveillance systems and a further seven were not representative of all LB cases (e.g. occupational exposures only). The remaining four papers described retrospective database reviews of general practice or hospital data. An Austrian study reported a mean national hospitalised LB incidence for 2005–2018 of 11.3 per 100,000 person-years. A Finnish study reported an incidence of 120 per 100,000 population in 2014. An Italian study reported an incidence for a single hospital in Verona during 2015–2019 of 0.99 per 100,000 inhabitants. Finally, a Dutch study reported national incidence during 2015–2019 ranging from 111 to 131 per 100,000 person-years.

Of the four SLRs identified, one reported the incidence of LB in children only. The other three reported sources of incidence data for European countries and noted substantial heterogeneity in study designs, populations sampled and case definitions that restricted data comparability.

**Added value of this study**

The Burden of Lyme Disease (BOLD) study is the first prospective study to comprehensively describe and evaluate the incidence of LB in high endemic sites across Europe using active surveillance and uniform methods, including case definitions and standardised laboratory testing, and thus minimising bias when comparing data from different countries. BOLD was conducted in a diverse group of European countries to increase the representativeness of results for endemic areas.

**Implications of all the available evidence**

In endemic areas of Europe, BOLD found higher LB incidence than reported from surveillance data for each of the six countries. The prospective, active surveillance and uniform methodology likely produced more accurate, comparable incidence estimates than previous studies. By clarifying disease burden, these data may guide future regional prevention efforts, including vaccination. The observed between-country heterogeneity in LB incidence emphasises the need for thorough monitoring and reporting of cases using standardised methods.

## Introduction

Lyme borreliosis (LB), caused by infection with bacteria from the *Borrelia burgdorferi* sensu lato species, is the most prevalent tick-borne disease in Europe. Early infection consists of a localised erythema migrans (EM) red skin rash or lesion which, if left untreated can be followed by a disseminated infection that affects the nervous system, joints and/or the heart.^1^ LB is endemic in many European countries, with approximately 24% of the European population residing in areas of high LB incidence. Risk areas continue to expand, a concerning trend that underscores LB as an important public health challenge.^2^ LB incidence has been reported for individual European countries through both active public surveillance and case estimation studies. However, despite consensus case definitions being developed by the European Union Concerted Action on LB (EUCALB), LB case definitions have been inconsistently implemented.^3^, ^4^ LB surveillance commonly under-ascertains LB cases. For example, Brestrich et al. estimated that 2·7 times more annual LB cases occurred in Norway, Finland, and Poland than were reported by surveillance in these countries.^5^ In addition, not all European countries conduct LB surveillance, and those that do can have substantial differences in both case definitions and methodologies, hindering multi-country incidence comparisons.^6^

Different combinations of laboratory tests are used to diagnose LB in different countries and within different clinical practices, impacting diagnosis, case confirmation, treatment decisions, and reporting.^7^ Detection of serum antibodies to *B*. *burgdorferi* consequent to infection is currently the diagnostic method of choice in a routine clinical setting, with a recommended 2-tier testing approach using a screening assay (such as an enzyme immunoassay [EIA]) followed by an immunoblot (such as a Western blot, dot blot, or microarray).^8^, ^9^ This forms part of many current European laboratory testing guidelines for LB,^10^ although a wide range of single and two-tier combinations of EIA and immunoblots with varying performance characteristics are used, and consequently there is a lack of standardisation across countries.^11^ Beyond serological tests, other tests for direct detection of *B*. *burgdorferi* sensu lato include bacterial culture, polymerase chain reaction (PCR), and sequencing-based methods from skin biopsies, cerebrospinal fluid (CSF), plasma, or synovial fluid.^12^, ^13^ These direct detection methods allow identification of genotype and serotypes for which *Borrelia afzelii* and *Borrelia garinii* account for most LB cases in Europe.^14^ However, while these tests enhance the overall sensitivity and specificity for diagnosis of Lyme borreliosis, these methods generally fall outside of the standard of care and are rarely used in the course of normal clinical practice.

The lack of multi-country epidemiological studies in Europe using uniform case definitions (including standardised laboratory testing) hinders accurate evaluation of LB as a public health problem, including the value of future prevention methods such as vaccination. The Burden of Lyme Disease (BOLD) study, was a prospective epidemiological study using active surveillance to measure medically attended LB. The study was conducted using standardised case definitions to better characterise LB burden at high endemic sites across Europe to assist in the identification of potential trial sites for assessment of a candidate outer surface protein A (OspA) Lyme disease vaccine. This paper reports results of the incidence analysis from the BOLD study, showing LB incidence rates across endemic areas in 6 central and northern European countries: the Czech Republic, Germany, Poland, Slovakia, Slovenia, and Sweden, determined using local standard of care as well as standardised study laboratory testing. Comprehensive understanding of LB incidence in Europe will enhance the understanding of the LB burden and may inform clinical trials and public health interventions to prevent LB. Further, the distribution of OspA in-silico types (IST) identified by molecular testing was determined.^15^

## Methods

### Study design and setting

The detailed BOLD study methodology has been published elsewhere,^16^ and relevant details are summarised in Fig. 1a. Briefly, this prospective, active surveillance, epidemiological study was conducted at 14 general practices/primary care practices located in areas of high LB incidence in 6 European countries: the Czech Republic (3 sites), Germany (5 sites), Poland (2 sites), Slovakia (2 sites), Slovenia (1 site), and Sweden (1 site). One site in Germany, as well as the Slovenian and the Swedish sites, each reported data collected from 2 to 4 practices. For Poland, while the BOLD methodology paper^16^ counted 3 sites separately according to Polish law, 2 were combined for the purpose of the BOLD analysis, as one site was the referral site of the other.

**Fig. 1:**
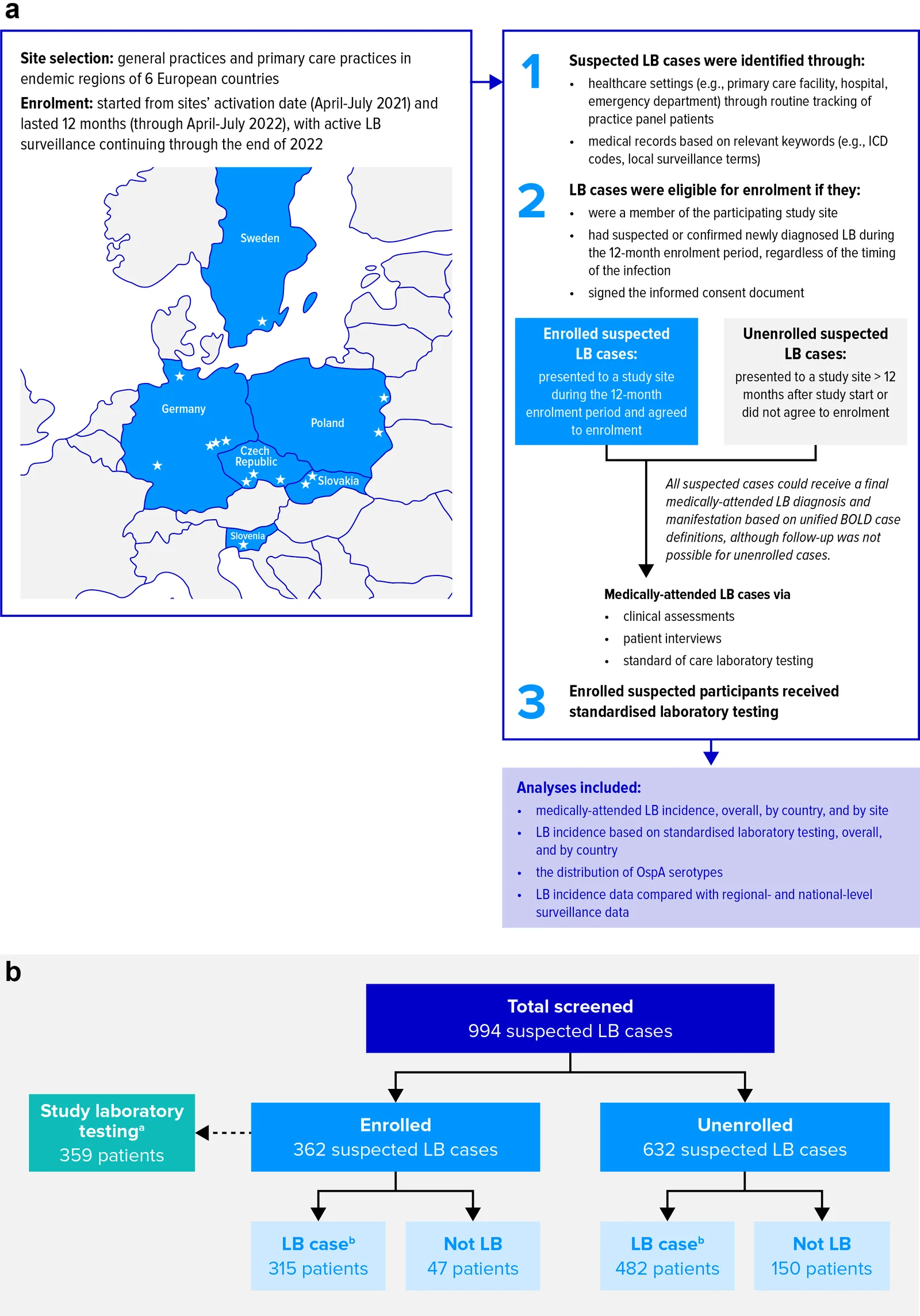
An overview of the (a) Burden of Lyme Disease study design and (b) participant disposition. Footnotes: BOLD = Burden of Lyme Disease; ICD = International Statistical Classification of Diseases; LB = Lyme borreliosis. ^a^198 of the 359 (55%) were positive by at least 1 test (MTTT, PCR, or culture). ^b^All LB cases fulfilled the study case definition (including standard of care testing for disseminated cases).

Selected study sites were primary care clinics with adequate research infrastructure to conduct vaccine clinical trials, and with appropriate primary care practice panels for incidence calculations. Study sites also had a historical incidence of LB (with or without laboratory-confirmation) of at least 500 per 100,000 population per year. At least 1 site in each country provided care to children, except Slovakia. Active surveillance for LB cases with enrolment of suspected or confirmed LB cases began following each site's activation date (April–July 2021) and lasted 12 months (through April–July 2022). Active surveillance without the opportunity for enrolment continued until the end of 2022. Study laboratory samples were collected from enrolled participants with suspected LB at study visit 1 (date of suspected diagnosis), visit 2 (28 days after suspected diagnosis) and visit 3 (10 months after suspected diagnosis). Standard of care (SOC) laboratory testing was performed at the discretion of the treating physician, and results were documented for enrolled participants at all study visits.

This study was approved by the local ethics committee for each participating site^16^ and was conducted in accordance with legal and regulatory requirements, the International Ethical Guidelines for Biomedical Research Involving Human Participants, the International Council for Harmonisation Good Clinical Practice Guideline, and the Declaration of Helsinki. Written informed consent was obtained for each participant before starting study-specific activities.

### LB cases

This study identified medically attended LB cases (referred to here as ‘LB cases’), comprising participants who sought medical care at their participating clinic and received a clinical diagnosis for EM or a clinical and SOC laboratory diagnosis for disseminated disease, according to the BOLD case definitions (developed from EUCALB definitions)^4^, ^16^ (Table S-1). All sites agreed to use the BOLD case definitions during the study period, and study-site personnel received standardised training on screening and diagnosis of LB based on clinical best practices and maintained a weekly log to support case identification. During active surveillance, suspected LB cases were identified through routine tracking of general practice and primary care contacts or through medical records. To minimise missed cases, efforts were made to identify cases occurring outside the participating site, e.g. sites reached out to local hospitals and emergency departments. To be offered study enrolment, cases had to be members of the participating study site, have newly identified suspected or confirmed LB during the 12-month enrolment period and have given informed consent; there were no exclusion criteria. Participants who did not enrol either presented to a study site with suspected or confirmed LB outside the 12-month enrolment period for each site or declined participation (reason not recorded). All participants had data available on demographics (Table S-2) and could receive a LB case diagnosis based on the BOLD case definitions (Table S-1). Enrolled cases also had medical, symptom, and care histories and were asked to complete a risk factor questionnaire.

Furthermore, all enrolled suspected LB cases underwent standardised laboratory testing (‘study laboratory testing’) to ensure laboratory testing practices were not a source of between-site variation in incidence. These results were only available for the enrolled cases and were not available to research sites until the end of the study.

### Study laboratory testing

All enrolled participants underwent study blood sample collection for serology testing at visits 1 to 3. Two 2 mm skin punch biopsies were obtained from any potentially LB-related skin rash in patients who consented. Any residual cerebrospinal fluid (CSF) and/or synovial fluid (SF) specimens from standard of care procedures were scavenged and sent to a central laboratory for testing. A participant was considered to be study laboratory confirmed for LB if they had a positive result at either visit 1 or 2 (acute phase). One of the skin biopsy samples and CSF were tested directly by molecular testing to confirm the presence of *B. burgdorferi* sensu lato and the associated OspA serotype (no synovial fluid samples were available). The parallel skin biopsy samples were cultured for *B. burgdorferi* sensu lato organisms, and isolates were subsequently typed by molecular testing. Molecular analyses were conducted in 2 steps, with an initial real-time (q) PCR test for the *Borrelia* 16S ribosomal RNA gene.^17^ Positive samples were then assessed by next-generation sequencing of the *ospA* gene, with analysis of the output sequence using the OspA IST bioinformatic pipeline as a proxy for serotype.^15^ Because the IST numbering corresponds to the serotype numbering for serotypes 1–8, the serotype nomenclature is used in this manuscript. Serological diagnostic testing was conducted using a custom modified two-tier testing (MTTT) immunodiagnostic algorithm composed of a first tier BioPlex 2200 Lyme Total assay from BioRad and a second tier VlsE1/pepC10 IgG/IgM enzyme-linked immunoassay (ELISA) from Zeus Scientific.^18^ Frozen sera, CSF, and flash frozen skin biopsy samples were stored at ‍< −80 °C and then sent to Pfizer laboratories in Pearl River, New York (United States) for analysis. Skin biopsies for culture were transported in BSK-II media supplemented with antibiotics at ambient temperature to Amsterdam Medical Centre (Netherlands) where they were incubated for up to 12 weeks to confirm the presence of spirochaetes.

### Data analyses

Estimation of incidence excluded data from one site in Poland because patients presenting with a tick bite were prophylactically treated with antibiotics even if there was no evidence of illness. Study size was based on feasibility, not on hypothesis testing as this was a descriptive study. Analysis was conducted using Statistical Analysis System (SAS) software (version 9.4).

### LB incidence

To support LB incidence calculations, a retrospective survey was conducted at each participating site to obtain the number of patients registered and the number of medically attended LB cases (using ICD-9 and ICD-10 codes and keywords) during 2019 and 2020 (Table S-3).

The population-based medically-attended incidence of LB cases per 100,000 population per year (identified through active surveillance) was calculated for years 2021 and 2022 using the practice panel population of each site. Confidence intervals were calculated assuming a Poisson distribution (Table S-4). Because surveillance began following site activation in April–July 2021, sites had a partial year of surveillance for the 2021 incidence calculation. To estimate the number of LB cases in 2021, the average proportion of LB cases that occurred each month in 2019 and 2020 (as reported in the practice panel survey) was calculated, and the available 2021 data for each site were increased proportionately to accommodate the missing months of the year. The 2019 and 2020 practice panel data were available by age group and sex, allowing calculation of incidence by these characteristics.

The proportion of study laboratory confirmed cases was only available for enrolled cases, but was assumed to be consistent between participants who enrolled and those who did not and was therefore extrapolated for all cases by country (separately for EM and disseminated cases). The incidence of study laboratory confirmed cases was then calculated for all participants for the 12-month enrolment period following each site's initiation. Since EM cases do not typically receive laboratory testing in real-world practice, a ‘combined incidence’ was also calculated, combining the medically-attended EM incidence (where no laboratory diagnosis was required), and the study laboratory incidence for disseminated cases.

To explore under-ascertainment of LB cases in surveillance data, the incidence of BOLD LB cases from 2021 to 2022 was compared to population-based LB incidence as reported by public health surveillance.

### Role of the funding source

This analysis was supported and jointly funded by Valneva and Pfizer Inc. as part of their co-development of a Lyme Disease vaccine. Pfizer Inc. was the Regulatory sponsor of the study, and was involved in the study design, data collection, data analysis, interpretation of data, the writing of the report, and the decision to submit the paper for publication.

## Results

### Disposition and participant summary

Of 994 suspected LB cases identified during the study period, 797 (80·2%) fulfilled the BOLD case definition (Fig. 1b, Table S-1): 94 from the Czech Republic, 63 from Germany, 30 from Poland, 173 from Slovakia, 95 from Slovenia, and 342 from Sweden (Table S-2). The mean age of the 797 LB cases was 53·2 years (standard deviation [SD], 18·6), 436 (54·7%) were female, and all 559 who reported their race were White. There were 615 (77·2%) cases diagnosed with an EM manifestation, 147 (18·4%) had Lyme arthritis (LA), 4 (0·5%) had Lyme neuroborreliosis, 2 (0·3%) had Lyme carditis, and 20 (2·5%) had an LB diagnosis without established LB clinical manifestations as defined in Table S-1 (e.g. systemic symptoms only); 9 (1·1%) presented with more than one manifestation. Manifestation results are presented in more detail in a separate BOLD publication.^19^ Laboratory diagnostics were conducted according to standard of care at each site. Although EM cases are typically clinically diagnosed, some countries nevertheless performed serological testing (Czech Republic 10/24, Germany 26/33, Poland 8/8, Slovakia 28/28, Slovenia 0/20, Sweden 0/97). Disseminated cases received SOC laboratory confirmation in-line with the BOLD case definition (with rare exceptions, for example where an EM was also present) (Figs. S-3 and S-4). No synovial fluid specimens were obtained, and no skin biopsies were performed for routine SOC laboratory diagnostics.

During the 12-month enrolment period, 362 individuals with suspected LB agreed to enrol in the BOLD study, and 315/362 (87·0% of all suspected enrolled participants) were diagnosed as medically-attended LB cases. Compared to all 797 LB cases, the enrolled LB cases had a slightly higher mean age (enrolled LB cases, 54·5 years [SD, 16·6]; all LB cases, 53·2 years [SD, 18·6]), a slightly higher proportion identifying as female (enrolled LB cases, 56·8%; all LB cases, 54·7%), and a higher proportion of disseminated cases (enrolled LB cases: EM 66·7%, disseminated 33·3%; all LB cases: EM 77·2%, disseminated 22·8%) (Table S-2).

### LB incidence

The overall incidence of medically-attended LB cases at BOLD study sites (excluding one site in Poland) was 657 per 100,000 population in 2021 and 661 cases per 100,000 population in 2022 (Table 1 and Fig. 2a). Slovakian sites had the highest incidence of LB cases in both 2021 and 2022, with 3059 per 100,000 population in 2021 (SK-1: 4366; SK-2: 1805 per 100,000) and 1860 per 100,000 population in 2022 (SK-1: 2938; SK-2: 826 per 100,000). The Swedish site had the second-highest incidence, with 1541 per 100,000 population) in 2021 and 1737 in 2022. In 2021 the Polish site had the lowest incidence (209 per 100,000 population), and in 2022 the German sites had the lowest incidence (75 per 100,000 population; range 0–143 per 100,000). Incidences from the 2019 and 2020 retrospective site survey can be found in Table S-3.

**Table 1:** Incidence of all medically-attended Lyme borreliosis cases per 100,000 population at study sites.

Countries and sites	Practice panel size	2021^a^				2022			12-mth enrolment period^b^		
		Cases	Annualised cases c	Incidence	95% CIs	Cases	Incidence	95% CIs	Cases	Incidence	95% CIs
All sites^c^	66,453	357	436	656·5	596, 721	439	660·6	600, 725	441	663·6	603, 729
Czech Republic sites	9484	33	36	376·3	263, 522	61	643·2	492, 826	35	369·0	257, 513
CZ-1	4032	15	18	438·7	259, 696	44	1091·3	793, 1465	15	372·0	208, 614
CZ-2	1309	4	4	305·6	83, 782	5	382·0	124, 891	4	305·6	83, 782
CZ-3	4143	14	14	337·9	185, 567	12	289·6	150, 506	16	386·2	221, 627
German sites	22,590	46	53	236·6	177, 309	17	75·3	44, 120	50	221·3	164, 292
DE-1	3549	4	7	205·2	85, 418	4	112·7	31, 289	7	197·2	79, 406
DE-2	4687	11	12	261·0	135, 453	0	0·0	0, 79	11	234·7	117, 420
DE-3	8368	16	18	213·5	127, 338	8	95·6	41, 188	17	203·2	118, 325
DE-4	3887	6	6	166·9	64, 354	2	51·5	6, 186	6	154·4	57, 336
DE-5	2099	9	10	456·4	215, 851	3	142·9	29, 418	9	428·8	196, 814
Poland site^d^, ^e^											
PL-1	5520	9	12	209·4	107, 370	20	362·3	221, 560	14	253·6	139, 426
Slovakia sites	4032	98	123	3058·8	2542, 3648	75	1860·1	1463, 2332	107	2653·8	2175, 3207
SK-1	1974	71	86	4365·9	3494, 5392	58	2938·2	2231, 3798	77	3900·7	3078, 4875
SK-2	2058	27	37	1805·1	1270, 2484	17	826·0	481, 1323	30	1457·7	984, 2081
Slovenia site^e^											
SL-1	13,771	21	42	303·5	219, 411	74	537·4	422, 675	53	384·9	288, 503
Sweden site^e^											
SE-1	11,056	150	170	1541·5	1319, 1791	192	1736·6	1500, 2000	182	1646·2	1416, 1904

CZ = Czech Republic; DE = Germany; LB = Lyme borreliosis; PL = Poland; SK = Slovakia; SL = Slovenia; SE = Sweden.

Note: Comparisons of incidence between sites should be made with caution given the variation in practice panel sizes.

a Year allocated based on date of LB diagnosis.

b Active surveillance for LB cases with enrolment began following each site's activation date (April–July 2021) and lasted 12 months (through April–July 2022).

c Annualised for months before study start based on proportion of LB cases from the corresponding calendar months in prior years 2019 and 2020. Annualisation was not needed for 2022 data because active surveillance was conducted for the entire calendar year.

d Excluding 1 site in Poland.

e This country only includes one study site, so the country's total values are the same as the single study site.

**Fig. 2:**
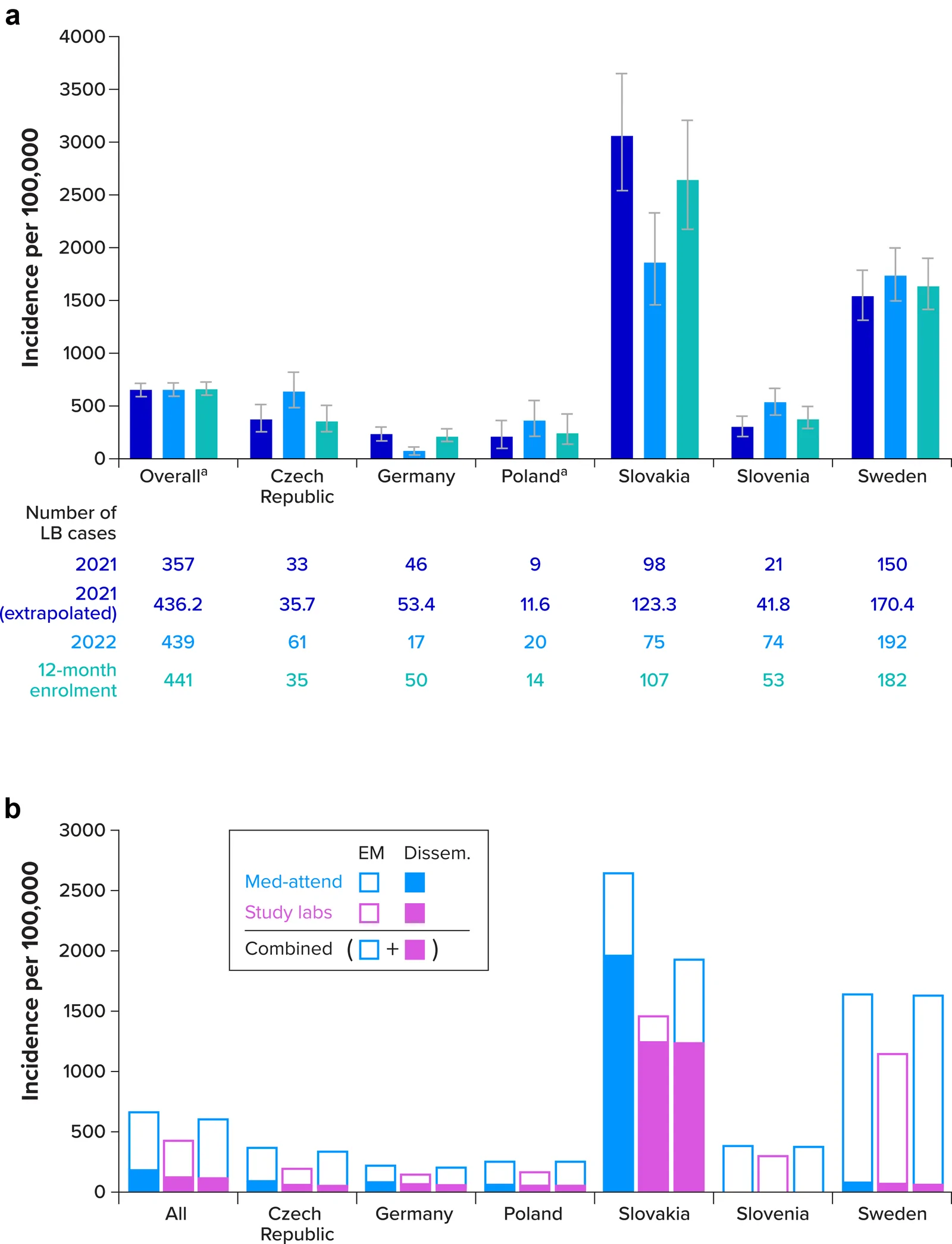
Incidence of (a) all medically attended Lyme borreliosis cases in 2021, 2022 and the 12-month enrolment period per 100,000 persons by country of study site with 95% confidence intervals, and (b) medically attended enrolled Lyme borreliosis cases, study laboratory confirmed enrolled Lyme borreliosis cases, and a combination (medically attended erythema migrans cases and study laboratory confirmed disseminated cases), for the 12-month enrolment period. Footnotes: LB = Lyme borreliosis. ^a^Excluding 1 site in Poland. Notes: Case counts for January–July 2021 (Fig. 2a) are based on annualised data. The combined estimated incidence bar (Fig. 2b) reflects the incidence that could be expected if study laboratory testing was performed in a real-world setting, with clinical diagnosis of EM cases (medically attended using the BOLD case definitions) and standardised laboratory confirmation of disseminated cases (study laboratory confirmed). The presentation of LB cases (EM and disseminated) by country is discussed in greater detail in the BOLD sister manuscript.^19^

The incidence of LB cases in the participating sites was highest in patients aged 45–64 years and ≥65 years (Fig. S-1). Incidence was also higher in females than in males in all sites except in Slovakia (Fig. S-2). When compared with population-based incidence as reported by public health surveillance, the incidence of LB was consistently higher at the BOLD study sites than the incidence of surveillance-reported LB cases, although there was substantial variation in the size of the multiplier between regions and countries (Table S-4).

### Study laboratory testing

Of the 362 enrolled suspected LB cases identified during the 12-month enrolment period, 359 received study laboratory testing. Serum samples were obtained from 358 cases, skin biopsies from 86 cases (all 86 were tested by PCR; 82 were cultured), and CSF from 1 case (tested by PCR). Of the 359 cases, 272 were tested by serum MTTT only; 81 were tested by MTTT, PCR, and culture; 5 were tested by MTTT and PCR (including 1 PCR on CSF); and 1 was tested by PCR and culture. Overall, 198 of the 359 (55%) were positive by at least 1 test (MTTT, PCR, or culture); 171 (48%) were positive by 1 test, 24 (7%) were positive by 2 tests, and 3 (0·8%) were positive by all 3 tests (Fig. 3, Fig. S-3). Results were positive in 190 of the 358 (53·1%) suspected LB cases tested by MTTT serology, 32 of 86 suspected LB cases (37·2%) tested by PCR, and 6 of 82 suspected LB cases (7·3%) tested by skin biopsy culture (Table S-5). Concordant and discordant study laboratory results between PCR, MTTT serology, and culture are shown in Table S-6. Data on seroconversion and seroreversion over the course of the study are presented in Supplementary Materials 1. The data on seroconversion are limited since approximately half of the participants were already seropositive at their first study visit. For seroreversion, 46/144 (31·9%) of participants who had been positive at visit 1 had seroreverted by month 10.

**Fig. 3:**
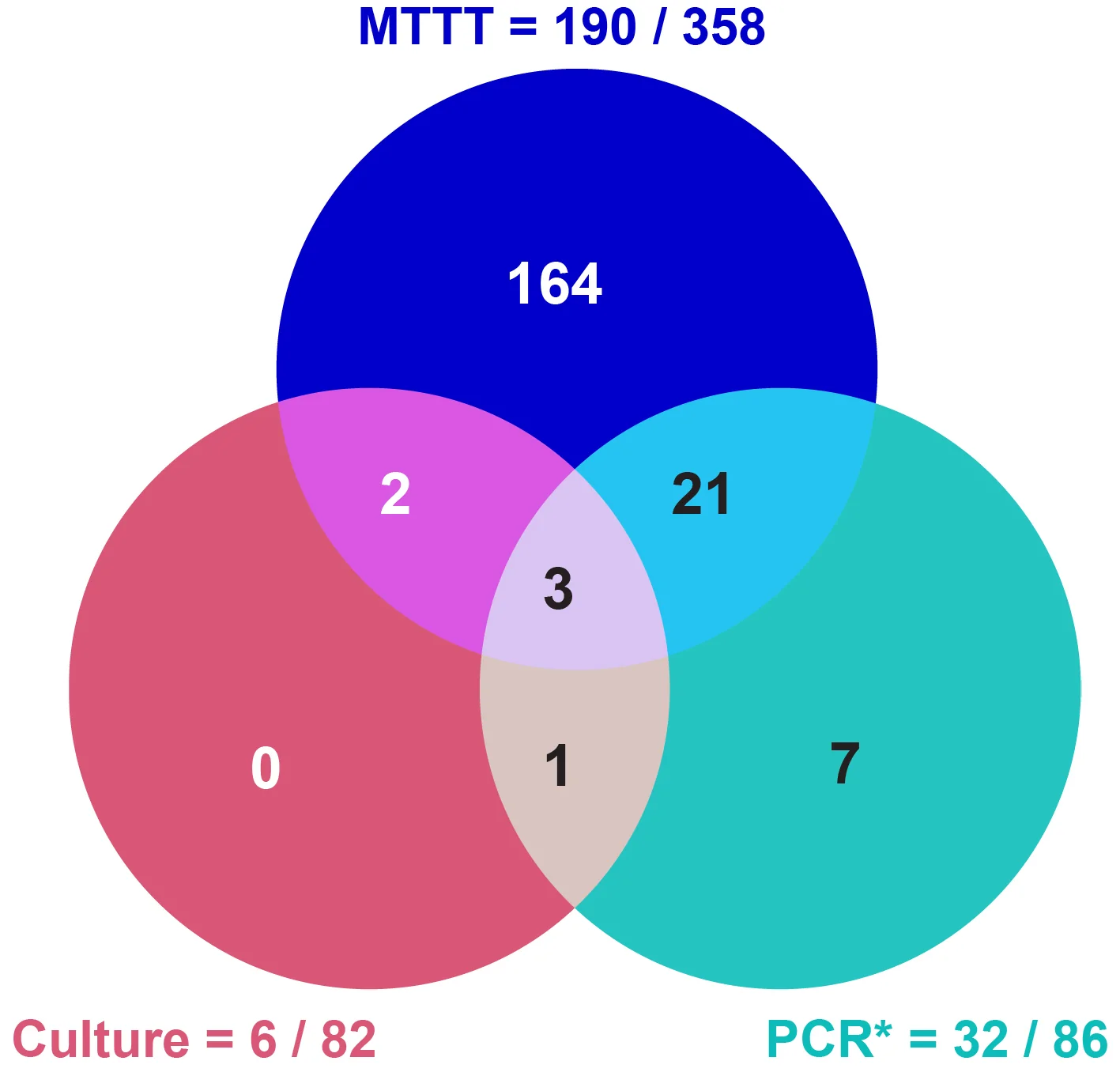
Venn diagram of study samples and testing (all positive tests for enrolled, suspected Lyme borreliosis cases [N = 359ˆ]). Footnotes: BOLD = Burden of Lyme Disease study; MTTT = modified two-tier testing; PCR = polymerase chain reaction. Note: Of the 190 participants with positive MTTT results, 1 case was diagnosed as not Lyme borreliosis by investigators. ˆThree (of 362) enrolled, suspected LB cases did not receive study laboratory testing. ∗16S PCR.

Among the 315 medically attended LB cases, 197 (62·5%) received positive study laboratory results. Of the 210 EM cases, 207 (98·6%) received study laboratory testing and 128 (61·8%) of these had positive results (Czech Republic n = 12/24 [50%], Germany n = 20/32 [63%], Poland n = 4/8 [50%], Slovakia n = 9/28 [32%], Slovenia n = 16/20 [80%], Sweden n = 67/95 [71%]). All of the 105 medically attended disseminated LB cases received study laboratory testing, and 69 (65·7%) were positive (Czech Republic n = 5/8 [63%], Germany n = 7/9 [78%], Poland n = 1/1 [100%], Slovakia n = 50/79 [63%], Slovenia n = 0/1 [0%], Sweden n = 6/7 [86%]). Of the 36 that tested negative in MTTT, 10 had a positive 2-tier SOC test, two tested positive in an EIA and a BLOT at separate visits, and a further four had a positive 1-tier BLOT. Two cases were diagnosed based on EM rash (a Slovenian case with multiple EMs, and a Swedish case with both EM and LA manifestations) and received no SOC testing. The remaining 18 cases were diagnosed as positive based on a 1-tier EIA test, and 17 of these cases were LA cases from Slovakia. Slovakian LA cases are discussed further in a BOLD sister publication.^19^ Medically-attended and study laboratory incidence of all LB cases per 100,000 population for the 12-month enrolment period is shown in Fig. 2b. As expected, the study laboratory incidence was lower than the medically attended incidence for every country, with an overall incidence of 414·2 per 100,000 study laboratory confirmed LB cases compared to 663·6 per 100,000 medically attended LB cases. When the ‘combined’ incidence is calculated (using medically attended incidence for EM and study laboratory confirmed incidence for disseminated cases), the overall incidence was 603·8 per 100,000. All countries except Slovakia had a ‘combined’ incidence that was at least 90% of their medically-attended incidence indicating consistency between the SOC and study laboratory testing for disseminated LB: Czech Republic 91%, Germany 92%, Poland 100%, Slovakia 73%, Slovenia 98%, Sweden 99%.

Of the 86 cases with an EM manifestation and skin biopsies tested by culture or PCR, *B. burgdorferi* sensu lato isolates from 34 (39·5%) were positive and further sequenced to determine *B. burgdorferi sensu lato* genospecies and serotypes. One isolate from Sweden gave an indeterminate result (the *Borrelia* genus was present, but the sample did not produce enough good sequence to determine an OspA serotype). *B. afzelii* (serotype 2) was the predominant genospecies/serotype identified in skin biopsies from 30 of 33 (90·9%) cases. These study participants came from Sweden (n = 14), Slovenia (n = 10), the Czech Republic (n = 3), Germany (n = 2), and Poland (n = 1). *B. garinii* serotype 6 was identified in the biopsies from 2 cases, 1 from Poland and 1 from Sweden. The biopsy from 1 German case was *B. garinii* serotype 8.^15^

## Discussion

The BOLD study is the first prospective epidemiological study of medically attended LB spanning multiple countries in Europe. Its high quality, standardised methods allowed for more accurate assessments of incidence in endemic regions of individual countries, and for more cross-country comparisons of such regions, when compared with previous studies which had varying study methodologies that hindered comparisons. Historically, differences in public health surveillance and diagnostic practices and assays have made it difficult to distinguish true differences in LB incidence (due to differences in healthcare access,^20^ awareness,^21^ environmental changes,^22^ or changes in tick populations^23^) from differences due to heterogeneity in surveillance methodology. Moreover, all previous studies have used passive surveillance systems or retrospective designs, which can lead to substantial under-ascertainment of incidence. By using a multinational design with standard LB case definitions and surveillance methodologies as well as clearly defined populations (i.e. the practice panels of primary care providers) and standardised study laboratory testing with greater sensitivity and specificity than conventional Lyme disease testing (including a more sensitive MTTT format and direct detection of the bacteria), the BOLD study provides data that are likely to reflect true incidences as well as true differences between the endemic regions of participating countries.

The incidence of medically attended LB in BOLD-participating general practices was 657 per 100,000 population in 2021 and 661 per 100,000 population in 2022. The incidence of LB was highest at the sites in Slovakia, followed by the Swedish site. The high incidence at the Swedish site is not unexpected; a previous publication on incidence in southern Sweden identified an increasing trend of LB over time and a LB rate of 1300/100,000 population in 2019 in Western Kronoberg.^24^ In contrast, the extremely high incidence in Slovakia is harder to explain, with an incidence which is much higher than a recent systematic literature review reported,^25^ and an unusual presentation of cases. Specifically, Slovakia reported a higher proportion of LA cases compared with other countries, a quarter of which (19/76 [25%]) received 2-tier testing as standard of care (or 28/76 [37%] when an EIA and a BLOT at separate study visits is included as 2-tier testing), with a further 10 receiving a 1-tier BLOT and 38 only receiving a 1-tier EIA (Figs. S-4 and S-5). However, when ‘combined’ incidence was calculated for the enrolled population based on medically-attended EM cases and study laboratory confirmed disseminated cases, the incidence at the Slovakian sites remained higher (1935 per 100,000 population) than any other BOLD country (Fig. 2b), indicating that differences in standard of care laboratory testing alone cannot explain the high incidence observed. The Slovakian LA cases are reviewed in depth in a sister publication on BOLD study LD case manifestations and symptoms.^19^

Incidence estimates in BOLD varied both between countries and between sites within a country (e.g. German sites recorded incidences ranging from 166·9 to 456·4 per 100,000 in 2021). This can be influenced both by varying exposure to the vector and by healthcare practices. Incidence also varied between years of the study, but not consistently. For instance, between 2021 and 2022 incidence doubled for a site in the Czech Republic (CZ-1) but reduced to about a third at the German sites overall. Both the spatial and temporal distribution of LB is highly dependent on the distribution of the tick vector, *Ixodes ricinus*. Studies have shown an association between LB incidence and tick abundance,^26^ the latter fluctuating as a result of a complex interplay of different environmental factors such as climate and rodent abundance.^27^ Additionally, the strains causing LB in Europe may vary between countries, which can also affect disease burden.^28^ It should also be noted that high-incidence sites were deliberately selected for the BOLD study, and it is possible that 2021 data includes participants with disseminated disease who had delayed seeking a diagnosis for LB in 2020 as a consequence of the COVID-19 pandemic. For example, Slovakian investigators reported practices were closed until shortly before the BOLD study started in April 2021.

Of the 198 cases that tested positive in study laboratory tests, 190 did so by MTTT serology and 32 by PCR. Neither test alone was sufficient to identify all cases. The six cases diagnosed by culture all received additional positive results by MTTT serology or PCR. Only 86 cultures/PCR samples could be collected from skin biopsies where an EM lesion was present. The low number of skin biopsy samples may be due to the invasive nature of biopsies, which patients often choose to avoid, preferring less invasive testing methods when possible. Of the skin biopsies obtained, 7·3% of culture biopsies were positive compared with 37·2% of PCR biopsies (Table S-5). Recovery by culture was lower relative to detection by PCR, as has been reported in other studies,^28^ potentially due to low concentration of the *Borrelia* in the epidermis and the difficulty in identifying the best location to sample. It is possible that uptake of antibiotics by study participants prior to culture samples being obtained may have resulted in a lower proportion of positive culture tests. Of the 82 culture biopsies taken, 34 (41·5%) had started antibiotics before their biopsy date. Most biopsies were collected from Slovenia and Sweden, where both had a high proportion of EM cases (Fig. 2b). Of the 34 *Borrelia* isolates that could be sequenced, 30/33 [91%] genospecies identified were *B. afzelii*, consistent with previous literature indicating that *B. afzelii* is most commonly associated with EM and other skin manifestations.^28^ The very low number of non-*afzelii* isolates in this prospective study that spans multiple countries highlights the challenges facing any serotype-specific evaluation (such as serotype-specific vaccine efficacy).

This study had several limitations. Study sites were selected in high-endemic areas and incidence described for the sites in each country cannot be extrapolated to the country as a whole, especially where a country (Poland, Slovenia and Sweden) had only one study site. Sites were specifically recruited to the study based on whether they had access to a primary care ‘practice panel’ to identify denominators for incidence calculations, but patients at one or more sites in the study may nonetheless not be fully representative of the populations of their local areas. Rates of tick-borne diseases are dependent on the underlying ecological cycles of their vector and can vary year-to-year. This study captured LB incidence in two years, and any interpretation beyond those years should be made with caution. Pre-season baseline serology specimens from the study participants were not available, so we could not determine whether serology-positive results were reflective of previous infections prior to study start. No synovial fluid specimen were obtained from suspected LA cases, probably because the study was set in GP practices. There was potential for misdiagnosis, especially of EM cases where a laboratory confirmation was not required (commercial ELISA tests have only approximately 54% sensitivity for EM cases^29^). Asymptomatic infections could not be routinely captured. The study was initiated as the COVID-19 pandemic was waning and healthcare-seeking behaviour will have been modified. As a consequence it is possible that there was greater under-reporting of cases in surveillance conducted in 2021 and 2022. Also, while we strove to capture all LB events in all settings, some patients with suspected disease might have been referred to clinical sites outside the selected study sites (e.g. hospitals) and thus not captured. To minimise this, sites were encouraged to identify events from other settings via routine methods for tracking the healthcare contacts of the practice panel patients, which was monitored through regular follow-ups with site personnel.

This manuscript has explored the incidence of LB in Europe, the primary objective in the BOLD study. BOLD had multiple secondary and exploratory objectives, the data for which will be published elsewhere; data on the manifestations and symptoms of LB have been published in a sister publication,^19^ and publications on Patient Reported Outcomes and Post-Treatment Lyme Disease Syndrome will be published separately.

### Conclusions

Using standardised methods to collect data from clinics located in high-endemic regions of six European countries, we report incidence estimates for LB that likely reflect true differences across the endemic regions of Europe rather than differences in surveillance methodology, and demonstrate wide heterogeneity in risk. The heterogeneity in LB incidence shows the difficulties inherent in aggregating estimates of LB incidence across regions, highlighting the need for thorough regional monitoring and reporting. It points also to high incidence foci at many sites across Europe; public health interventions for the prevention of LB should be considered.

## Contributors

KH, ALB, BDG, FJA, AP, EB and JS designed and coordinated the study. AMM, FS, JSB and the BOLD study group enrolled participants into the study. WVK, KTS, MC, AvD, JH and RS conducted diagnostics. KH, ALB, JSB, YT and JE accessed and verified the data. YT oversaw the statistical analysis. All authors interpreted the data, reviewed the final version of the manuscript, and approved the manuscript for submission. All authors confirm that they had full access to all the data in the study and accept responsibility to submit for publication.

## Data sharing statement

Upon request, and subject to review, Pfizer will provide the data that support the findings of this study. Subject to certain criteria, conditions and exceptions, Pfizer may also provide access to the related individual de-identified participant data. See https://www.pfizer.com/science/clinical-trials/trial-data-and-results for more information.

## Editor note

The Lancet Group takes a neutral position with respect to territorial claims in published maps and institutional affiliations.

## Ethics

This study was approved by all sites’ local ethics committees (Appendix B)^16^ and was conducted in accordance with legal and regulatory requirements, the International Ethical Guidelines for Biomedical Research Involving Human Participants, the International Council for Harmonisation Good Clinical Practice Guideline, and the Declaration of Helsinki. Written informed consent was obtained for each participant before the start of study-specific activities.

## Declaration of interests

KH, ALB, YT, WVK, KTS, MWC, FJA, JE, AP, RS, EB and JHS are employees of Pfizer Inc. and may hold shares or stock options in the company. RS is an inventor on patents related to Lyme disease vaccines. BDG was an employee of Pfizer during his involvement with the BOLD study, currently consults with Pfizer and Minervax, received support from Sinovac to attend a congress, and may hold shares or stock options in Pfizer Inc. AM-M gave a lecture for Pfizer and took part in research grants from Pfizer and DiaSorin related to tick-borne and respiratory diseases. FS served on the scientific advisory board for Roche on Lyme disease serological diagnostics and on the scientific advisory board for Pfizer on Lyme disease vaccines and is an unpaid member of the steering committee of the ESCMID Study Group on Lyme Borreliosis/ESGBOR. JWH and APV work for AMC, which received funding from Pfizer to complete culturing and related laboratory work for the BOLD project. JWH has ongoing research collaborations with Pfizer, Inc., and collaborates, or has collaborated, with Moderna, Antigen Discovery, Inc., Bio-Rad Laboratories, Abbott, and ZEUS Scientific on other projects on Lyme borreliosis. JWH has an application for a provisional patent related to Borrelia antigens pending. JSB reports no conflicts of interest.
