# Supplementary material for: Incidence of medically attended Lyme borreliosis across endemic regions of six European countries (BOLD): a prospective multicentre active surveillance study
Source: Lancet Reg Health Eur. 2026 Oct 26;72:101907. doi: 10.1016/j.lanepe.2026.101907 (PMC13734844; doi:10.1016/j.lanepe.2026.101907)
Supplement: Supplementary file 1 [file mmc1.docx]

# Supplementary

Table S-1. BOLD case definitions

| Presentation ^a^ | Sign/Symptom | Laboratory Diagnostic(s) ^b^ |
| --- | --- | --- |
| Erythema migrans | Characteristic red or bluish-red patch, with or without central clearing | Positive IgG/IgM on serum Bbsl antibody testing OR  Positive PCR of Bbsl result from biopsy OR  Positive culture of Bbsl from biopsy |
| Borrelial lymphocytoma | Painless bluish-red nodule or plaque, usually on ear lobe, ear helix, nipple, or scrotum |  |
| Acrodermatitis chronica atrophicans | Long-standing red or bluish-red lesions, usually on the extensor surfaces of extremities. Initially doughy swelling. Possible skin induration and fibroid nodules over bony prominences |  |
| Lyme neuroborreliosis | Meningo-radiculoneuritis (Bannwarth syndrome), facial palsy, meningitis, encephalomyelitis, OR cerebral vasculitis | Intrathecal Bbsl IgM and/or IgG antibodies OR  Positive intrathecal *Borrelia* antibody index (CSF versus serum) reflecting intrathecal Bbsl antibody production OR  Positive PCR of Bbsl result from CSF OR  Positive culture of BbsI from CSF |
| Lyme carditis | Acute onset of high degree atrioventricular conduction disturbances, rhythm disturbances, myocarditis, OR pancarditis | Positive IgG/IgM on serum Bbsl antibody testing |
| Lyme arthritis | Marked swelling in 1 or few large joints, most often the knee | Positive IgG/IgM on serum Bbsl antibody testing OR  Positive PCR of Bbsl result from synovial fluid or tissue OR  Positive Culture of BbsI from synovial fluid or tissue |
| LB ocular manifestations | Conjunctivitis, uveitis, papillitis, episcleritis, OR keratitis | Positive IgG/IgM on serum Bbsl antibody testing OR  Positive PCR of Bbsl result from ocular fluid OR  Positive culture of BbsI from ocular fluid |

Bbsl = Borrelia burgdorferi sensu lato; BL = borrelial lymphocytoma; BOLD = Burden of Lyme Disease; CSF = cerebrospinal fluid; EM = erythema migrans; IgG = immunoglobulin G; IgM = immunoglobulin M; LB = Lyme borreliosis; PCR = polymerase chain reaction.

^a^ All presentations were considered disseminated Lyme manifestations and required laboratory confirmation, except for EM (singular lesion) and BL; EM or BL was only considered disseminated if there were multiple lesions and laboratory confirmation. Other EM cases could be diagnosed based on clinical symptoms alone. Disseminated cases that presented with more than one manifestation including an EM rash could be diagnosed clinically without laboratory confirmation.

^b^ At least one positive standard of care diagnostic test result was required for laboratory confirmation of medically attended LB.

Table S-2. Demographics and clinical characteristics of medically attended LB cases

|  | All cases  (N = 797) | Enrolled cases  (N = 315) |
| --- | --- | --- |
| **Country, n** |  |  |
| Czech Republic | 94 | 32 |
| Germany | 63 | 42 |
| Poland | 30 | 9 |
| Slovakia | 173 | 107 |
| Slovenia | 95 | 21 |
| Sweden | 342 | 104 |
| **Sex, n (%)** |  |  |
| Female | 436 (54·7) | 179 (56·8) |
| Male | 361 (45·3) | 136 (43·2) |
| **Age, years** |  |  |
| Mean (SD) | 53·2 (18·6) | 54·5 (16·6) |
| Median (Q1, Q3) | 57 (42, 67) | 57 (45, 67) |
| **Race, n ^a^** |  |  |
| White | 559 | 315 |
| Asian | 0 | 0 |
| Not reported ^b^ | 238 | 0 |
| **Manifestations, n (%)** |  |  |
| Erythema migrans | 615 (77·2) | 210 (66·7) |
| Lyme neuroborreliosis | 4 (0·5) | 2 (0·6) |
| Lyme arthritis | 147 (18·4) | 80 (25·4) |
| Lyme carditis | 2 (0·3) | 1 (0·3) |
| Other ^c^ | 20 (2·5) | 13 (4·1) |
| > 1 Manifestation ^d^ | 9 (1·1) | 9  (2·9) |

LB = Lyme borreliosis; N/A = not applicable; Q1 = quartile 1; Q3 = quartile 3; SD = standard deviation.

^a^ No individuals had Black or African American race or were multiracial.

^b^ One site in Sweden did not have access to information on race for participants who did not enrol.

^c^ Other = LB diagnosis without established LB clinical manifestation as defined in Table S-1, e.g., systemic symptoms only.

^d^ Erythema migrans and Lyme arthritis (n = 6), erythema migrans and Lyme carditis (n = 1), erythema migrans and Lyme neuroborreliosis (n = 1), and Lyme arthritis and pronounced unspecific general symptoms (n = 1).

Table S-3. Incidence per 100,000 population per year of medically attended LB cases overall, by country sites and by study site, 2019 and 2020

|  |  | 2019 ^a^ | | 2020 ^a^ | |
| --- | --- | --- | --- | --- | --- |
| Countries and sites | Practice panel size | Incidence per 100,000 at BOLD sites | 95% CI^b^ | Incidence per 100,000 at BOLD sites | 95% CI^b^ |
| **All sites ^b^** | 63,569^c^ | 1,033·5 | 956, 1116 | 1,025·7 | 948, 1108 |
| Czech Republic sites | 9,484 | 1,223·1 | 1011, 1467 | 1,001·7 | 810, 1225 |
| CZ-1 | 4,032 | 2,182·5 | 1751, 2689 | 1,661·7 | 1288, 2110 |
| CZ-2 | 1,309 | 611·2 | 264, 1204 | 611·2 | 264, 1204 |
| CZ-3 | 4,143 | 482·7 | 295, 746 | 482·7 | 295, 746 |
| Germany sites | 19,706^c^ | 629·2 | 523, 750 | 527·8 | 431, 640 |
| DE-1 | 3,549 | 140·9 | 46, 329 | 169·1 | 62, 368.0 |
| DE-2 | 4,687 | 725·4 | 502, 1014 | 832·1 | 592, 1138 |
| DE-3 | 8,368 | 621·4 | 464, 815 | 358·5 | 242, 512 |
| DE-4 | 1,003^c^ | 1,595·2 | 912, 2591 | 797·6 | 344, 1572 |
| DE-5 | 2,099 | 809·9 | 472, 1297 | 1,000·5 | 619, 1529 |
| Poland site ^d^  PL-1 | 5,520 | 688·4 | 487, 945 | 760·9 | 548, 1029 |
| Slovakia sites | 4,032 | 1,339·3 | 1006, 1748 | 1,314·5 | 985, 1719 |
| SK-1 | 1,974 | 2,077·0 | 1491, 2818 | 1,773·0 | 1235, 2466 |
| SK-2 | 2,058 | 631·7 | 336, 1080 | 874·6 | 518, 1382 |
| Slovenia site  SL-1 | 13,771 | 406·7 | 307, 528 | 915·0 | 762, 1089 |
| Sweden site  SE-1 | 11,056 | 2,433·1 | 2151, 2742 | 2,098·4 | 1837, 2387 |

CZ = Czech Republic; BOLD = Burden of Lyme Disease; DE = Germany; LB = Lyme borreliosis; PL = Poland; SK = Slovakia; SL = Slovenia; SE = Sweden.

^a^ Retrospective practice-reported counts based on ICD-9 and ICD-10 codes.

^b^ Confidence interval assumes a Poisson distribution.

^C^ The practice panel size for site DE-4 was lower in 2019/2020 than in 2021/2022 because retrospective data came from the main site only, whilst the prospective study also included data from two satellite sites.

^d^ Excluding 1 site in Poland.

Table S-4. Incidence per 100,000 population per year of medically attended LB cases from BOLD, and surveillance-reported LB cases from public health surveillance^a^, 2021 and 2022

|  |  | 2021 | | | 2022 | | |
| --- | --- | --- | --- | --- | --- | --- | --- |
| Country/region (state) | BOLD study sites | Country/regional incidence | Incidence at BOLD study sites (95% CI) ^a,b^ | Ratio | Country/regional incidence | Incidence at BOLD study sites (95% CI)^a^ | Ratio |
| **Czech Republic** |  | 27·0 | 376·3 (263, 522) | 13·9 | 32·7 | 643·2 (492, 826) | 19·7 |
| South Moravia | CZ-1 | 17·9 | 438·7 (259, 696) | 24·5 | 17·2 | 1,091·3 (793, 1465) | 63·4 |
| South Bohemia | CZ-2/3 | 42·0 | 330·2 (196, 522) | 7·9 | 83·0 | 311·8 (182, 499) | 3·8 |
| Germany |  | 32·2 ^c^ | 236·6 (177, 309) | 7·3 | 27·4 ^c^ | 75·3 (44, 120) | 2·7 |
| Hesse | DE-1 | N/A | 205·2 (85, 418) | N/A | N/A | 112·7 (31, 289) | N/A |
| Schleswig-Holstein | DE-2 | N/A | 261·0 (135, 453) | N/A | N/A | 0·0 (0, 79) | N/A |
| Saxony | DE-3/4/5 | 47·4 | 236·4 (164, 331) | 5·0 | 41·8 | 90·6 (48, 155) | 2·2 |
| Poland |  | 32·8 | 209·4 ^d^ (107, 370) | 6·4 | 45·9 | 362·3 ^d^ (221, 560) | 7·9 |
| Podlaskie | PL-1 | 61·4 | 209·4 ^d^ (107, 370) | 3·4 | 81·2 | 362·3 ^d^ (221, 560) | 4·5 |
| Slovakia |  | 11·4 | 3,058·8 (2542, 3648) | 268·3 | 25·4 | 1,860·1 (1463, 2332) | 73·2 |
| Trencin | SK-1/2 | 7·6 | 3,058·8 (2542, 3648) | 402·5 | 81·9 | 1,860·1 (1463, 2332) | 22·7 |
| Slovenia |  | 139·2 | 303·5 (219, 411) | 2·2 | 190·0 | 537·4 (422, 675) | 2·8 |
| Central | SL-1 | 125·5 | 303·5 (219, 411) | 2·4 | 166·1 | 537·4 (422, 675) | 3·2 |
| Sweden |  | N/A | 1,541·5 (1319, 1791) | N/A | N/A | 1,736.6 (1500, 2000) | N/A |
| Blekinge county | SE-1 | N/A | 1,541·5 (1319, 1791) | N/A | N/A | 1,736.6 (1500, 2000) | N/A |

CZ = Czech Republic; BOLD = Burden of Lyme Disease; DE = Germany; LB = Lyme borreliosis; N/A = not applicable; PL = Poland; SK = Slovakia; SL = Slovenia; SE = Sweden.

^a^ The following data sources were used. Czech Republic: <https://szu.gov.cz/knihovna-szu/casopisy/seznamy-odebiranych-casopisu/rok-2021/>; Germany: <https://survstat.rki.de/Default.aspx>; Poland: <https://wwwold.pzh.gov.pl/oldpage/epimeld/index_p.html>; Slovakia: <https://www.epis.sk/InformacnaCast/Publikacie/VyrocneSpravy.aspx>; Slovenia: <https://nijz.si/wp-content/uploads/2023/09/Spremljanje-nalezljivih-bolezni-ki-jih-prenasajo-clenonozci-v-Sloveniji-v-letu-2021.pdf> and [Spremljanje-nalezljivih-bolezni-ki-jih-prenasajo-clenonozci-v-Sloveniji-v-letu-2022.pdf](https://nijz.si/wp-content/uploads/2024/03/Spremljanje-nalezljivih-bolezni-ki-jih-prenasajo-clenonozci-v-Sloveniji-v-letu-2022.pdf). Sweden has no public surveillance for LB.

^a^ All LB cases.

^b^ Year allocated based on date of LB diagnosis.

^c^ Incidence derived from case numbers reported by Robert Koch Institute divided by population data for the 9 states that conduct public health surveillance for LB.

^d^ Excluding 1 site in Poland.

Table S-5. Study Laboratory Testing for All Enrolled, Suspected Lyme Borreliosis Cases

| Specimen (test) | Result | All enrolled, suspected LB cases (N = 362),  n (%) ^a^ | Medically attended LB  (n = 315),  n (%) ^b^ | Not LB (n = 47),  n (%) ^c^ |
| --- | --- | --- | --- | --- |
| **Study laboratory results** | |  |  |  |
| Serum (MTTT) | Positive | 190 (53·1) | 189 (60·8) | 1^d^ (2·1) |
|  | Negative | 168 (46·9) | 122 (39·2) | 46 (97·9) |
|  | Not done | 4 (n/a) | 4 (n/a) | 0 (n/a) |
| Skin biopsy (16S PCR) | Positive | 32 (37·2) | 32 (37·2) | 0 (–) |
|  | Negative | 54 (62·8) | 54 (62·8) | 0 (–) |
|  | Not done^e^ | 276 (n/a) | 229 (n/a) | 47 (n/a) |
| Skin biopsy (culture) | Positive | 6 (7·3) | 6 (7·3) | 0 (–) |
|  | Negative | 76 (92·7) | 76 (92·7) | 0 (–) |
|  | Not done^f^ | 280 (n/a) | 233 (n/a) | 47 (n/a) |
| CSF (EIA, immunoblot, or 16S PCR) | Positive | 0 (–) | 0 (–) | 0 (–) |
|  | Negative | 1^g^ (100) | 1^g^ (100) | 0 (–) |
|  | Not done^h^ | 361 (n/a) | 314 (n/a) | 47 (n/a) |

CSF = cerebrospinal fluid; EIA = enzyme immunoassay; EM = erythema migrans; LB = Lyme borreliosis; MTTT = modified 2-tier testing; N/A = not applicable; PCR = polymerase chain reaction.

^a^ Among 358 tested samples.

^b^ Among 311 tested samples.

^c^ Among 47 tested samples.

^d^ Case received a positive MTTT result, reported nonspecific systemic symptoms, and received a diagnosis of “not LB” by the primary physician.

^e^ There were 218 participants with an EM manifestation in total (including those with >1 manifestation). Of the 276 suspected LB participants where a skin biopsy PCR was not done, 132 had an EM manifestation and could have had a skin biopsy taken.

^f^ There were 218 participants with an EM manifestation in total (including those with >1 manifestation). Of the 280 suspected LB participants where a skin biopsy culture was not done, 136 had an EM manifestation and could have had a skin biopsy taken. Only 82/86 skin biopsies were cultured because 4 skin biopsy culture samples were not taken due to logistical issues (a Swedish site ran short of culture media).

^g^ Taken from a suspected Lyme neuroborreliosis case and tested negative on PCR, although the case tested positive overall on MTTT serology.

^h^ CSF testing was dependent on availability of residual SOC samples, and a CSF sample would not be taken for the purpose of the study. CSF samples were only expected for neuroborreliosis cases (n= 3).

Table S-6. Comparison of Study Laboratory Results for Medically Attended Lyme Borreliosis Cases with a skin biopsy result

| Culture versus 16S PCR results (n = 82 ^a^) | | |
| --- | --- | --- |
|  | Positive PCR | Negative PCR |
| Positive culture | 4 | 2 |
| Negative culture | 25 | 51 |
| Culture versus serology results (n = 81 ^b^) | | |
|  | Positive serology | Negative serology |
| Positive culture | 5 | 1 |
| Negative culture | 50 | 25 |
| 16S PCR versus serology results (n = 85 ^c^) | | |
|  | Positive serology | Negative serology |
| Positive PCR | 24 | 8 |
| Negative PCR | 34 | 19 |

Colour key: Green shows concordant results and orange shows discordant results.

 CSF = cerebrospinal fluid; PCR = polymerase chain reaction.

One participant had a negative PCR result from a CSF sample and is not included in the results shown here.

^a^4 participants had a PCR skin biopsy result (3 positive, 1 negative) but no culture sample.

^b^230 participants had a serology result (134 positive, 96 negative) but no culture sample; 1 participant had a culture result (negative) but no serology taken.

^c^225 participants had a serology result (130 positive, 95 negative) but no PCR sample; 1 participant had a PCR result (negative) but no serology taken.

Materials 1. Seroconversion and seroreversion

Seroconversion was defined as a change from negative in MTTT at visit 1 to positive at visit 2 or 3. Seroreversion was defined as a change from positive in MTTT at visit 1 to negative at visit 2 or 3.

Results: Among medically attended LB cases, 263 of 315 (83·5%) had MTTT serology results at both visit 1 and visit 2; the remaining 52 either declined a blood draw or did not have a visit 2 (if the participant was ≥21 days after LB diagnosis at Visit 1, then Visit 2 data collection was performed at Visit 1 and no separate Visit 2 was performed). Of the 263 participants with MTTT serology results at visit 1 and visit 2, 119 tested negative at visit 1 and could be assessed for seroconversion at visit 2; 17 of 119 (14·3%) participants seroconverted by visit 2, which occurred within a median of 28 days (range, 15–47 days) after visit 1. Furthermore, of the 102 participants who were negative at visit 1 and visit 2, 83 returned for visit 3; 2 of 83 participants (2·4%) seroconverted by visit 3, which occurred 331 and 341 days after visit 1. Of the 119 participants who could be assessed for seroconversion, 15 of 83 EM cases (18·1%) and 4 of 36 disseminated (11·1%) cases seroconverted. Similarly, of the 263 participants with MTTT serology results at visit 1 and visit 2, 144 (54·8%) tested positive at visit 1 and could be assessed for seroreversion at visit 2. By visit 2, 10 of 144 participants (6·9%) seroreverted with a median time of 22.5 days (range, 15-28 days) after visit 1. Furthermore, of the 134 participants who were positive at visit 1 and visit 2, 111 (82·8%) returned for visit 3; 36 of 111 (32·4%) seroreverted within a median of 320 days (range, 303-403 days) after visit 1. Of the 144 participants who could be assessed for seroreversion, 37 of 85 EM cases (43·5%) and 9 of 59 disseminated cases (15·3%) seroreverted.

Interpretation: The data on seroconversion are limited since participants who were already seropositive at their first study visit could not be included in the analysis. For seroreversion, 31.9% of participants who had been positive at visit 1 had seroreverted by month 10. The higher rate of seroreversion seen for EM cases (43·5%) compared with disseminated cases (15·3%) is consistent with existing literature that reports less robust antibody responses for EM cases. The seroreversion analysis is limited by the lack of pre-study serology, with patients potentially seropositive from previous *Borrelia* infections. Nearly all patients in BOLD were treated with antibiotics at their first visit, which may have affected both seroconversion and seroreversion. This could reduce antigenic stimulation, reduce seropersistence, and subsequently limit or delay antibody responses.

Figure S-1. Incidence of medically attended LB cases per 100,000 persons per year by age group and country of study site in the 12-month enrolment period

Case counts for 2021 (January-July) are based on annualised data. We did not observe a peak of LB in children; the reasons for this are unclear, but paediatric case numbers were low.

^a^ Excluding 1 site in Poland.

Figure S-2. Incidence of medically attended LB cases per 100,000 persons by sex and country of study site in the 12-month enrolment period

Case counts for 2021 (January-July) are based on annualised data.

^a^ Excluding 1 site in Poland.

Figure S-3. Venn Diagram of Study Testing (Enrolled, Suspected Lyme Borreliosis Cases [N = 362])

MTTT = modified two-tier test; PCR = polymerase chain reaction; CSF = cerebrospinal fluid

* 16S PCR

Figure S-4. Standard of Care 1- and 2-Tier Testing on Medically Attended Lyme Borreliosis Cases by Manifestation and Country

EM = Erythema migrans; Dis = disseminated; EIA = enzyme immunoassay; BLOT = Western blot, immunoblot, or microarray; LA = Lyme arthritis; LC = Lyme carditis.
Note: Two-tier testing was defined as an EIA and a BLOT test in the same study visit. Negative testing comprised a negative BLOT result (in Slovakia, n = 4) or a negative EIA (in Germany, n = 5; Poland, n = 3; Slovakia, n = 13). Of the 46 disseminated cases diagnosed by a SOC 1-tier EIA, 35 were diagnosed based on IgG (with or without IgM), and 11 were diagnosed by IgM only: Czech Republic n=1 (‘other’ manifestation), Slovakia n=9 (Lyme arthritis [LA]), Sweden n=1 (EM/Lyme carditis [LC]). Seven disseminated cases did not receive SOC laboratory testing: 1 multiple EM in Slovakia, 5 EM cases that also had a disseminated manifestation in Sweden, and 1 German case with fatigue which tested positive on study MTTT and was included under the BOLD case definitions (with manifestation = Other) following discussion with investigators. The disseminated cases in Slovakia were all LA with the exception of one LC (2-tier positive), one Lyme neuroborreliosis (1-tier EIA positive), and one case with both EM and LA (2-tier positive).

Figure S-5.	Type of Test Kit^a^ Used in Standard of Care Diagnoses of Enrolled, Suspected Lyme Borreliosis Cases (N = 362)

EIA = enzyme immunoassay.

NB Slovenia is not included in this figure because no SOC tested was performed.

^a^ Test kits used included Chemiluminescent Immunoassay, EIAs, Western blots, immunoblots, and microarrays manufactured by Diasorin, Biomedica, Euroimmun, Mikrogen, Viramed, and TestLine Clinical Diagnostics.
