# Supplementary material for: Incidence of medically attended Lyme borreliosis across endemic regions of six European countries (BOLD): a prospective multicentre active surveillance study
Source: Lancet Reg Health Eur. 2026 Oct 26;72:101907. doi: 10.1016/j.lanepe.2026.101907 (PMC13734844; doi:10.1016/j.lanepe.2026.101907)
Supplement: Supplementary file 2 [file mmc2.docx]

# Appendix A

## Investigators of the Burden of Lyme Disease (BOLD) study group

- Dr. Lenka Dybova; Doktor Brno, S.R.O.; Brno, Czech Republic
- Dr. Miroslav Pavlasek; ADMED, S.R.O.; Ceske Budejovice, Czech Republic
- Dr. Daniela Verdanova; Ordinace praktickeho lekare pro deti a dorost, Jindrichuv Hradec, Czech Republic
- Dr. Joachim Weimer; Private Practice of Dr. Joachim Weimer; Reinfeld, Germany
- Dr. Christine Kosch; Private Practice of Dr. Christine Kosch; Pirna, Germany
- Dr. Kai Mehltretter; Velocity Clinical Research; Leipzig, Germany
- Dr. Hans-Detlev Stahl; AmBeNet GmbH; Leipzig, Germany
- Dr. Viliam Cibik, MUDr; Viliam Cibik, PhD, S.R.O.; Pruske, Slovakia
- Dr. Dagmar Zakova, MUDr; Zakova, s.r.o; Ambulancia vnutorneho lekarstva, Trencin, Slovakia
- Dr. Barbara Nieradko-Iwanicka; Lubelskie Centrum Diagnostyczne, Swidnik, Poland and Medical University of Lublin, Lublin, Poland
- Dr. Dorota Bielska; Akademicka Praktyka Medycyny Rodzinnej, Bialystok, Poland

The BOLD study group also included Drs Berglund, Moniuszko-Malinowska, and Strle who are listed separately as co-authors.

# Appendix B

## Ethics committee approval information

| ***Country*** | ***Ethics Committee*** | ***Initial Approval Date*** | ***Initial Approval Code*** |
| --- | --- | --- | --- |
| Czech Republic | Eticka komise IKEM a FTN Fakultni Thomayerova nemocnice Videnska 800 Praha 4 - Krc, 140 59 | 16 February 2021 | 12/21+4217/21 |
| Germany | Ethik-Kommission bei der Landesärztekammer Hessen Hanauer Landstrasse 152 60314 Frankfurt am Main | 12 March 2021 | 2021-2414-zvBO |
| Germany | Ethikkommission bei der Ärztekammer Schleswig-Holstein Bismarckallee 8–12 23795 Bad Segeberg | 12 March 2021 | 034/21 m |
| Germany | Ethikkommission bei der Sächsischen Landesärztekammer Schützenhöhe 16 01099 Dresden | 12 March 2021 | EK-BR-22/21-1 |
| Poland | Komisja Bioetyczna przy Lubelskiej Izbie Lekarskiej w Lublinie ul. Chmielna 4 Lublin, 20-079 | 1 February 2021 | 140/2021/KB/VIII |
| Poland | Komisja Bioetyczna przy Uniwersytecie Medycznym w Bialymstoku ul. Jana Kilinskiego 1 Bialystok, 15-089 | 25 March 2021 | APK.002.169.2021 |
| Poland | Komisja Bioetyczna przy Okregowej Izbie Lekarskiej z siedziba w Bialymstoku ul. Swietojanska 7 Bialystok, 15-082 | 21 April 2021 | 12/2021/VIII |
| Slovakia | Eticka Komisia Trencianskeho samospravneho Karja K dolnej stanici 7282/20A Trencin, 911 01 | 10 February 2021 | No code provided—the approval references Pfizer’s protocol number: C4601002 |
| Slovenia | Komisija Republike Slovenije za medicinsko etiko Stefanova 5 Ljubljana, 1000 | 31 May 2021 | 0120-53/2021-4 |
| Sweden | Etikprövningsmyndigheten Box 2110 Uppsala, 750 02 | 5 May 2021 | 2021-00604 |
